# A Practical Treatise on Massage

**Published:** 1885-01

**Authors:** 


**A Practical Treatise on Massage.** Its History, Mode of Application and Effects, Indications and Contra-indications. With over fourteen hundred cases. By DOUGLAS GRAHAM, M. D., Fellow of the Massachusetts Medical Society. New York: William Wood & Co. 1884.

This work gives a history of massage, the mode of its application, its physiological effects, and the uses of massage in nervous exhaustion and anæmia of women, in uterine diseases, and the effects of massage upon internal organs; the central nervous system and its diseases in writers' cramp and other affections; neuralgia and peripheral paralysis, muscular rheumatism, sprains and joint affections, etc. The scope of the work is broad, and the author brings to his work a large experience and observation in the uses of this treatment in local and general diseases. We think its publication called for by the special attention now directed to massage in diseases not amenable to medication and the beneficial effects derived therefrom under skilled direction. We commend the work to those who are investigating the merits of friction, kneading, manipulating, rolling and percussion of the external tissues of the body, with a view to their curative, palliative or hygienic effects.

